# Getting a GRP on histone deacetylase inhibitor selectivity

**DOI:** 10.18632/oncotarget.20855

**Published:** 2017-09-13

**Authors:** Husheng Ding, Cristina Correia, Scott Kaufmann

**Affiliations:** Scott Kaufmann: Department of Molecular Pharmacology and Experimental Therapeutics and Division of Oncology Research, Mayo Clinic, Rochester, MN, USA

**Keywords:** HDAC inhibitor, RASGRP1, RAF1, BIM upregulation, BCL2 mutations

Histone deacetylase (HDAC) inhibitors [1] are FDA approved for the treatment of lymphoid malignancies, including cutaneous T cell lymphoma (romidepsin, vorinostat) and multiple myeloma (panobinostat). While these agents have also undergone extensive clinical testing alone and in combination with a variety of other agents in solid tumors, clinical activity compelling enough to lead to FDA approval has been elusive. Until now it has been unclear why HDAC inhibitors as a class seem to be more active in lymphoid malignancies than in other neoplasms.

Previous understanding of the action of HDAC inhibitors has been based on both the demonstration that they alter gene expression through their impact on histone acetylation [1] and the more recent recognition that they impact lysine acetylation of other cellular proteins, including heat shock protein 90 (HSP90). When HSP90 becomes lysine acetylated as a consequence of HDAC inhibition, it has been reported to release client proteins, leading to their degradation [2]. Additional studies have demonstrated that increased signaling through the mitogen-activated protein (MAP) kinase pathway can lead to HDAC inhibitor resistance [3]. How the effects of HDAC inhibitors on gene expression, MAP kinase signaling or HSP90 function leads to selective killing of malignant lymphoid cells has not been obvious.

**Figure 1:**
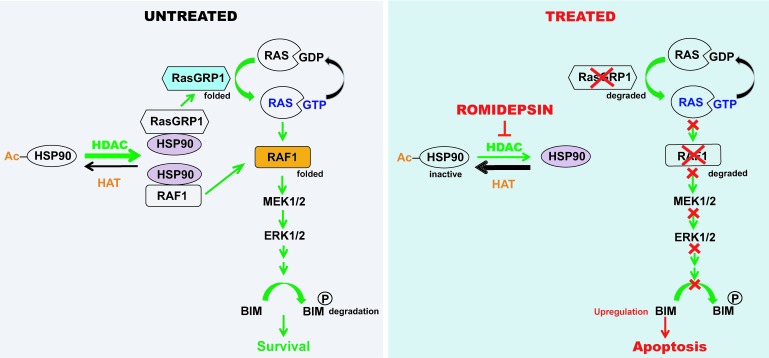
MAP kinase signaling in lymphoid malignancies in the absence (left) and presence (right) of the HDAC inhibitor romidepsin.

Recent studies by Ding et al. [4] provide new insight into this question as summarized in Fig. 1. Following up the observation that HDAC inhibitors upregulate the proapoptotic protein BIM [3-5], these investigators traced BIM upregulation, in part, to inhibition of MAP kinase signaling. Working their way upstream, Ding et al. identified two components of the MAPK pathway, the guanine nucleotide exchange factor RASGRP1 and the serine/threonine kinase RAF1, that are markedly downregulated in a variety of malignant human lymphoid lines by numerous HDAC inhibitors. Importantly, this downregulation occurred at the protein level without any substantive change in mRNA [4]. Further studies demonstrated that treatment with either HDAC inhibitors or the HSP90 inhibitor tanespimycin causes dissociation of HSP90 from RASGRP1 and RAF1, leading to degradation of both of these proteins. Particularly germane to the question of HDAC inhibitor selectivity, RASGRP1 is expressed almost exclusively in lymphoid cells [6], with higher levels in malignant T cells than in malignant B cells [7]. Without RASGRP1, RAS-mediated survival signaling is markedly diminished because of stabilization of the RAS•GDP complex [6].

These observations have potential implications not only for the mechanism of action of HDAC inhibitors, but also for overcoming drug resistance in lymphoid malignancies. Previous observations of Correia et al*.* [8] demonstrated that a subset of *BCL2* gene-rearranged lymphomas (follicular lymphoma and germinal center-type diffuse large B cell lymphoma) have amino acid altering *BCL2* mutations that, at least in some instances, are able to enhance the anti-apoptotic effects of the corresponding protein. The study by Ding et al. [4] reported that lymphoma cell lines with *BCL2* mutations are more resistant to DNA damaging treatments, including ionizing radiation, etoposide and cytarabine, than lymphoid lines without *BCL2* mutations. In contrast, *BCL2*-mutant and *BCL2*-wild type lines were equally sensitive to HDAC inhibitors, possibly reflecting the ability of the upregulated BIM to induce apoptosis whether *BCL2* is mutated or not.

Because the study of Ding et al. focused on sensitivity of malignant B cell lymphoma and did not examine cell lines derived from either peripheral T cell lymphoma or multiple myeloma, the two diseases for which HDAC inhibitors are currently approved, there is still more work to be done to determine whether the mechanism shown in Fig. 1 contributes to the high activity of HDAC inhibitors in these diseases. Nonetheless, the findings of Ding et al*.* have several implications. First, they suggest that high levels of RASGRP1 might be a potential biomarker for susceptibility of individual lymphoid neoplasms to HDAC inhibitors. Second, these findings suggest that further preclinical and possible clinical study of HDAC inhibitors might be beneficial in *BCL2*-mutant lymphoid malignancies that fail to respond to conventional therapy and/or BH3 mimetics. In short, further study of RASGRP1 as a determinant of sensitivity to inhibitors of both HSP90 and histone deacetylases might be a fruitful area of future investigation.
